# A qualitative study on rehabilitation services at primary health care: insights from primary health care stakeholders in low-resource contexts

**DOI:** 10.1186/s12913-024-11748-9

**Published:** 2024-10-23

**Authors:** Maria Y Charumbira, Farayi Kaseke, Thandi Conradie, Karina Berner, Quinette A Louw

**Affiliations:** 1 Division of Physiotherapy, Department of Health and Rehabilitation Sciences, Faculty of Medicine and Health Sciences, Stellenbosch University, Cape Town, South Africa; 2 Department of Rehabilitation Sciences, Faculty of Medicine and Health Sciences, University of Zimbabwe, Harare, Zimbabwe

**Keywords:** Low-resource settings, Primary health care, Rehabilitation, South Africa, Qualitative, Zimbabwe

## Abstract

**Background:**

The chasm between rehabilitation needs and available rehabilitation services is widening. In most low-resource contexts, there are inadequate rehabilitation professionals at primary health care (PHC), leaving nonrehabilitation primary care providers’ (PCPs) and district rehabilitation professionals and managers to address patients’ rehabilitation needs. This study explored rehabilitation and non-rehabilitation PCPs’ and managers’ perspectives on the situation of rehabilitation service provision in PHC settings and the challenges experienced in providing rehabilitation care.

**Methods:**

In this descriptive, exploratory qualitative study, individual semi-structured interviews and focus group discussions were conducted with purposefully sampled PCPs in Manicaland, Zimbabwe and Eastern Cape, South Africa. The transcripts were analysed thematically using Atlas.ti. version 22.2^®^.

**Results:**

Thirty-six PCPs (29 nonrehabilitation and 7 rehabilitation) and one district manager participated in the study. The current PHC rehabilitation services in the two low-resource contexts were described as inadequate, if not nonexistent. District rehabilitation professionals attempted to fill the gap through outreach and home visits, but resource limitations, particularly in Zimbabwe, often hampered this strategy. The nonrehabilitation PCPs took on task-shifting roles in the identification, referral, and education of patients with rehabilitation needs in the absence of rehabilitation professionals at PHC. Challenges encountered in providing rehabilitation care at PHC included unsupportive leadership, human resource shortages, lack of comprehensive PHC rehabilitation guidelines, no or delayed rehabilitation referral, lack of clear communication strategies, and users’ low demand for PHC rehabilitation.

**Conclusion:**

Tailored approaches, including context-specific rehabilitation guidance for existing task-shifting models, increased investment in rehabilitation and increased rehabilitation awareness, are needed to establish basic rehabilitation services in the described contexts because they are mostly absent. Importantly, the PHC systems in which rehabilitation is to be nested need to be strengthened.

**Supplementary Information:**

The online version contains supplementary material available at 10.1186/s12913-024-11748-9.

## Introduction

The chasm between rehabilitation needs and available rehabilitation services is widening. Rehabilitation is an integral component of comprehensive healthcare aimed at restoring or enhancing individuals’ functioning, independence, and quality of life [1]. Approximately one-third of the world’s population will require rehabilitation related to their health condition at some point in their lives [2]. However, less than half of individuals requiring rehabilitation in low- and middle-income countries (LMICs) can access the care they need [1]. This need is expected to increase as larger aging populations, surging chronic and noncommunicable diseases (NCDs) and persistent conflict-induced injuries are expected to contribute to an exponential increase in disability and functioning problems [3]. While rehabilitation services have traditionally been concentrated in specialized settings, such as hospitals or rehabilitation centres, there is increasing recognition of the need to extend these services at the primary health care (PHC) level to increase access to services and continuity of care across the life course [4]. This paradigm shift aligns with the principles of PHC, emphasizing accessibility, community-based care, and patient-centeredness (rather than disease-centeredness) [5].

Like many other LMICs, South Africa (SA) and Zimbabwe face significant challenges in the provision of rehabilitation services at PHC [1]. Although both countries have committed to PHC re-engineering for universal health coverage, evidence shows that rehabilitation services remain neglected [6]. Inadequate resources, infrastructure, and rehabilitation workforces pose barriers to equitable access to rehabilitation care, particularly in rural and underserved areas [1]. The training of rehabilitation professionals in both countries is modelled on the British system [7]. One university in Zimbabwe offers Bachelor degree programs in Occupational Therapy (OT), Speech and Language Therapy (SLT), Audiology and Physiotherapy (PT) as well as Masters in PT and Doctoral degrees in PT and OT [8]. Eight universities in SA offer at least four-year degree programs in OT and PT, seven of which offer bachelor’s degree training in SLT and audiology, and four offer three rehabilitation programs at the master and doctoral degree levels [9]. Despite these training programs, 3% of SA’s public healthcare rehabilitation workforce is working within PHC [10]. Most of these are recently graduated community service therapists and thus are largely inexperienced and unsupported in caring for the complex health needs of PHC patients [11], due to rehabilitation’s exclusion from SA’s PHC standard treatment guidelines [12]. These challenges are compounded by social determinants of health, including poverty, limited education, and cultural barriers, which impact individuals’ access to rehabilitation services and adherence to treatment plans [13]. Considering these challenges, available PCPs play a crucial role in ensuring access to and obtaining quality rehabilitation services for individuals who seek healthcare services at these grassroots levels and who may never enter the health system.

A recent retrospective review of PHC rehabilitation records in one SA metropolitan district revealed that clinic visits were the primary service delivery mode in addition to home visits and outreaches [14]. The study also identified PHC rehabilitation service delivery problems such as incomplete documentation of referrals and follow up of patients, and short intervention periods [14]. A few qualitative studies have explored the perceptions of rehabilitation professionals regarding the integration of rehabilitation at PHC [11, 15–17], and have further highlighted rehabilitation exclusion from mainstream healthcare. Yet only a limited number of rehabilitation professionals are found to be working at the PHC level in these settings [10]. This signifies an important gap in considering the perspectives of other PCPs (mostly nurses) [18], who have direct experience in delivering healthcare to the vulnerable populations, as well as the district hospital managers who oversee the PHC facilities. By engaging the voices of such PCPs and managers to inform rehabilitation and PHC policy and practice, we may gain an understanding of the targeted strategies required to promote equitable access and strengthen rehabilitation service delivery to benefit vulnerable and underserved populations in resource-constrained settings. This study explores the perspectives of PCPs and managers on the current situation of rehabilitation service provision in urban and rural PHC settings in SA and Zimbabwe and describes the challenges they encounter in providing rehabilitation care.

## Methodology

### Study design

This study used a descriptive, exploratory qualitative design to obtain new insights into the perspectives of PCPs and managers in the Manicaland province of Zimbabwe and Eastern Cape province of SA on current PHC rehabilitation service delivery. The phenomenological approach, situated within an interpretivist paradigm, was useful in understanding the subjective meanings that participants gave to their experiences and acknowledged the socially constructed reality of healthcare provision in these contexts resulting in multiple interpretations of events [19]. The exploratory nature of the study enabled the researchers to contribute to the development of new knowledge on the topic that is of special relevance to practitioners and policymakers since little is known about the status of PHC rehabilitation service delivery in low-resource contexts [19]. The use of individual interviews and focus group discussions aligned with the qualitative descriptive approach, facilitating a rich description of the participants’ perspectives and experiences in providing rehabilitation services within low-resource PHC settings. The report followed the standards for reporting qualitative research (SRQR) [20].

### Researcher characteristics and reflexivity

Qualitative research acknowledges that each researcher brings a unique background, experiences, values, perspectives, and social and professional identity, which can affect the research process. The researchers capitalised on personal, interpersonal, methodological and contextual reflexivity throughout the research process [21]. The primary investigator (PI) had nine years of clinical experience as a physiotherapist working in similar low-resource settings. Thus, the PI was familiar with the challenges and nuances of providing rehabilitation services in resource constrained PHC systems but acknowledged the changes that could have occurred over the ten years that she had moved to academia. While this background may have added to her understanding of the meanings behind participants’ accounts, it also required a critical appraisal of the researcher’s positionality on the data collection and analysis process to mitigate any preconceived assumptions or potential biases that may have affected the study results. The PI engaged in reflexive analysis through the documentation of her continual thoughts, reflections and methodological decisions on each phase of the research process in a reflexive journal and regularly collaborated with the research team through a peer-review process. Additionally, the PI acknowledged the power dynamics that existed during her interactions with some participants. The participants were reassured of their insights’ value to the study and consenting participants were engaged in a ‘member-checking’ process that ensured that the researchers’ interpretations accurately reflected the meaning behind the participants’ responses. Integrating these reflexive practices improved transparency and self-awareness in ensuring the study’s confirmability.

### Study setting

**Fig. 1:**
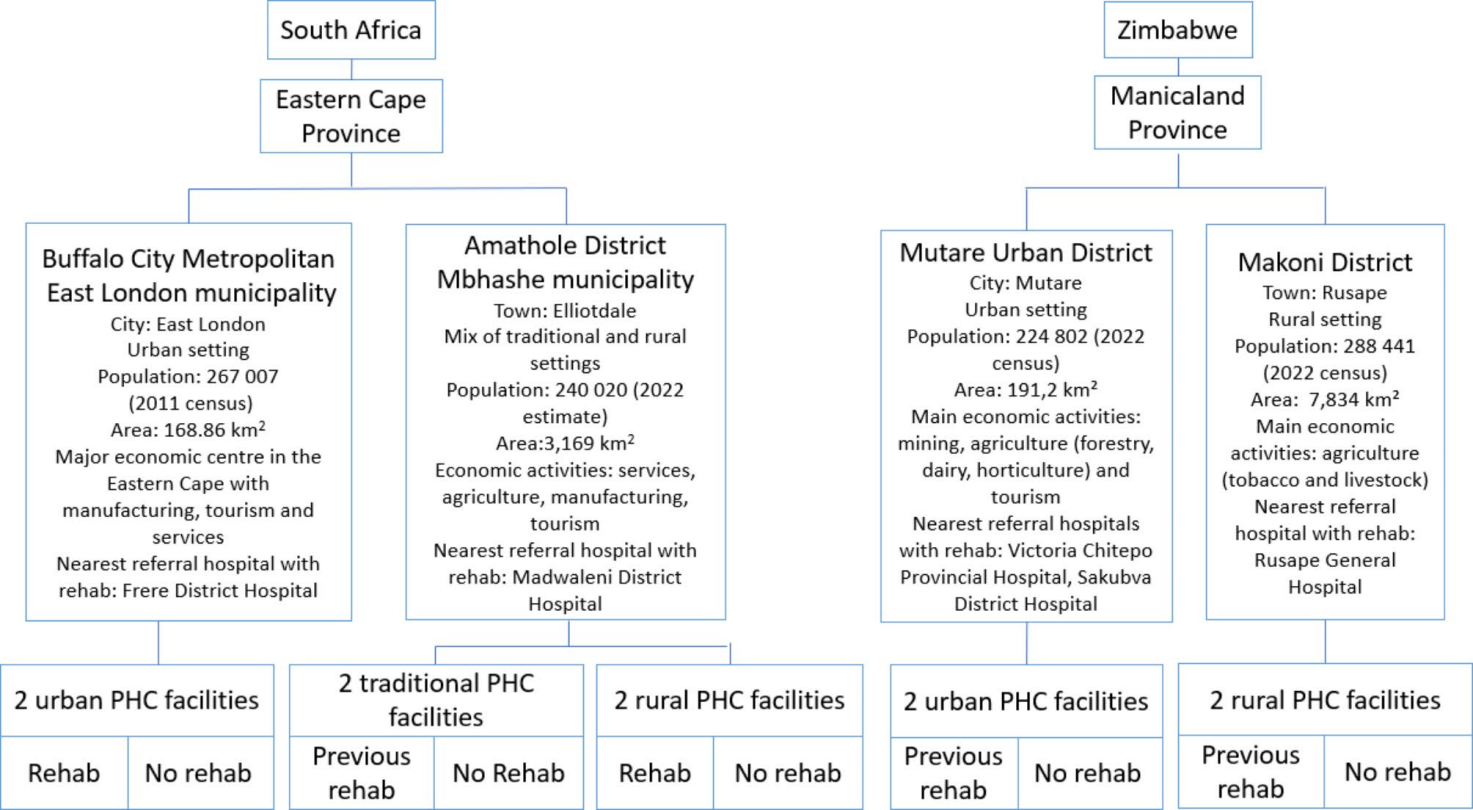
Overview of the study settings [24–27]

The study was conducted in 10 systematically selected PHC facilities in two districts of Manicaland (Mutare Urban and Makoni) and two districts of the Eastern Cape (Amathole and Buffalo City). These provinces are among the poorest in both countries, with the majority of the populations residing in rural areas [22, 23]. Within each district, one PHC facility was selected based on its geographical location (rural or urban) and whether it offered rehabilitation services (Fig. 1).

South Africa’s public PHC system consists of approximately 3 500 clinics and community health centres [28], while Zimbabwe has approximately 1 600 PHC facilities consisting of major clinics, polyclinics, mission clinics, city councils/municipal clinics, and rural health centres [29]. Both countries’ PHC systems are largely nurse-led, with medical officers in some PHC facilities [18]. The nurses and doctors serve as first point of contact for PHC patients and are often the gatekeepers to other specialist health services including rehabilitation. Community health workers (CHWs) also visit households to identify health risks and provide linkages between communities and PHC facilities [30]. The provision of primary rehabilitation services in both settings is largely dependent on the district health system. Thus, district hospital managers, including district medical officers (DMOs) and district nursing officers (DNOs), often oversee the resource allocation and operation of PHC facilities within the district. In Zimbabwe, rehabilitation technicians (RTs) undergo two years of training and work under the supervision of licenced therapists to assist in providing basic patient care and therapy services [31]. Since licenced rehabilitation therapists (OTs, PTs and SLTs) are rarely found at district hospitals, especially in rural remote areas, RTs are often found managing district rehabilitation departments and are expected to provide services at PHC by means of outreach. South Africa has a community service program that requires recently graduated therapists to serve for at least one year in underserved areas [11]. Hence, rural district hospitals are often served by community service therapists who are expected to provide outreach services to PHC facilities. Furthermore, SA community health centres are larger PHC facilities that may be equipped to provide outpatient rehabilitation services on an appointment basis.

### Participant sampling and recruitment

Systematic sampling was used to select one urban district and one rural district from SA and Zimbabwe. Within each district, one PHC facility was selected based on its geographical location (rural or urban) and whether it offered rehabilitation services. Purposive sampling was employed to identify PCPs with knowledge and at least six months of experience in PHC to provide informative responses [32]. Rehabilitation professionals (OTs, PTs, SLTs and RTs) from the district hospitals serving the selected PHC facilities were also included because they provided services at PHC. Additionally, district hospital managers (e.g., DMOs and DNOs) were included due to their administrative and managerial roles in overseeing the PHC facilities within the district hospitals’ catchment areas. A maximum variation approach was used to ensure representation across different professions, levels of experience, and geographical locations of PHC facilities. The participants were recruited verbally and then provided with written information about the study.

The CHWs were recruited through primary care nurses (PCNs) using the snowballing technique, which is also a purposeful non-probability method of sampling in qualitative research [33]. Snowball sampling was chosen because CHWs, who are integral to the PHC system in both South Africa, often operate in dispersed and informal networks that are not accessible through other sampling strategies. The PCNs referred initial participant CHWs who further recruited additional participants within their networks who had at least six months of experience in PHC.

Participant sampling continued until data saturation was achieved; no new insights emerged from additional interviews, and sufficient information was gathered to address the research questions [34].

### Data collection

One focus group discussion with 5 and 4 participants was held in Zimbabwe and SA, respectively. This method was discontinued due to a shortage of PCPs in most facilities, making it difficult to take participants away from their work at the same time. However, the data from the small focus groups were considered in this paper because the groups consisted of experienced participants and facilitated better understanding of the issues raised [35]. Therefore, between June 2021 and January 2022, the PI (MYC), who is experienced in qualitative research and conducting interviews, conducted individual face-to-face interviews and telephone interviews. All interviews were recorded electronically and lasted between 20 and 45 min. The interviews were conducted in the participants’ language of choice, primarily English, except for one focus group and one individual interview which were conducted in isiXhosa with the assistance of a professional interpreter. Research assistants (in SA, TC, a physiotherapist with experience in conducting qualitative research and in Zimbabwe, VNK, a nursing manager with qualitative interview experience) were present for quality assurance and notetaking. An interview guide (Supplementary file 1) was used to cover themes of interest with prompts and follow-up questions to elaborate on their responses. The development of the interview guide was informed by the aims and objectives of the study and literature on similar topics [16, 36, 37]. Some questions included the following: *During your consultations*,* how do you ensure that patients with disabilities or problems with day-to-day functioning receive rehabilitation? What rehabilitation services do you provide at this clinic (at PHC)? Can you share some insights into your experiences in ensuring that patients access and receive rehabilitation at PHC? Follow-up: Please relate any challenges and complexities that you encounter in doing so.* New relevant issues that arose during preceding interviews were subsequently addressed. For example, the reasons for discontinuation of outreach visits to some clinics, which were unknown to PCNs, were clarified by interviews with district rehabilitation professionals. No repeat interviews were needed. Member-checking was performed by clarifying the responses to ensure that the participants’ expressions were accurately understood. To facilitate honest responses, interviews were conducted in closed rooms at the PHC facilities, with nonparticipants prohibited from entering.

### Data processing and analysis

Professional transcribers translated and transcribed the recordings verbatim. The transcripts were returned to participants who provided initial consent to be recontacted via WhatsApp for verification. The data were managed using the computer-assisted qualitative data analysis (CAQDAS) software Atlas.ti. version 22.2^®^.

The interview transcripts were analysed using inductive thematic analysis [38]. The PI conducted the initial coding of the transcripts by repeatedly reading the transcripts to familiarize them with the data and then identified common conceptual subthemes and patterns emerging from the data under the assigned major themes. The notes that were taken by the research assistants were used to triangulate the data from the interview transcripts. After refining the subthemes, further detailed analysis of the data was performed in relation to the research objectives. Two senior authors (KB and QL) each read and coded three different transcripts. Several discussions of the preliminary coding were held by all the reviewers to share their perspectives and understanding of the participants’ accounts. Differences were resolved through discussion until a consensus was reached. A codebook was created, which was applied to the rest of the transcripts. Thus, an iterative process of identifying and adjusting code names and definitions, identifying and adjusting recurring concepts and patterns, and organizing them into subthemes and categories followed. Finally, a report was written by editing and situating the analysis in context.

### Techniques to enhance trustworthiness

Credibility was facilitated by employing purposive sampling and presenting the various viewpoints held by the participants. Investigator triangulation was utilized, with three researchers collaboratively making coding and analysis decisions based on a subset of transcripts. Transferability was enabled through detailed descriptions of the participants, contexts, and research methods, allowing readers to assess the study’s relevance to other contexts. Dependability was ensured by including exemplary quotations from most participants to substantiate the emerging themes. Member checking during interviews and the return of transcripts for participants to verify accuracy guarded against the influence of our biases on the analysis and interpretation of participants’ responses, thus ensuring confirmability. An audit trail was kept through meticulous documentation of the research process.

### Ethical considerations

All procedures performed in studies involving human participants were in accordance with the ethical standards of the institutional and/or national research committee and with the 1964 Helsinki Declaration and its later amendments or comparable ethical standards. All transcript data were anonymized by removing any names from the transcripts. All data generated from the study, including electronic recordings and identifying information, were kept in an access-restricted location. The study forms part of the main author’s PhD project [39], and approval was granted from the Stellenbosch University Health Research Ethics Committee (S21/01/002 (PhD)) and Medical Research Council of Zimbabwe (MRCZ/A/2916). The Permanent Secretary to the Ministry of Health, Provincial Medical Directorate of Manicaland Province, District Medical Officers, and PHC facility managers granted study permission in Zimbabwe. The Eastern Cape Department of Health, District Offices of the Department of Health and PHC facility managers granted study permission to participate in the SA. Written informed consent was obtained from all individual participants involved in the study before the interviews.

## Results

### Participant characteristics

Thirty-seven participants took part in the study. These included 29 nonrehabilitation PCPs, one district hospital manager and seven rehabilitation professionals (six from the district hospital but providing rehabilitation at PHC and one from PHC). Table 1 summarizes their demographic characteristics and duration of experience in PHC.

**Table 1:** Study participant characteristics

	South Africa	Zimbabwe	Total
Total participants	22	15	37
Urban	11	10	21
Rural	11	5	16
Median age (range)	31 (27–58)	45 (33–63)	42 (27–63)
Experience in PHC (mean (standard deviation))	5 (7.24)	16.7 (4.04)	9.8 (8)
Nurses	11	13	24
District nursing officer*	0	1	1
Primary care nurses	11	12	23
Nurse assistants	4	0	4
Doctor	1	0	1
Rehabilitation professionals*	5	2	5
Physiotherapists	2	0	2
Occupational therapists	1	0	1
Speech and language therapists	1	0	1
Rehabilitation technicians	0	2	2
Community health worker	1	0	1

*From district hospital (except one rehabilitation professional who was from a South African PHC facility)

Of these participants, nine attended the focus groups. The first focus group held in SA consisted of two female PCNs and two female nursing assistants aged between 42 and 58 years with years of PHC experience ranging from 1 to 10 years. The second focus group conducted in Zimbabwe consisted of five female PCNs whose age ranged from 42 to 55 years, while years of PHC experience ranged from 3 to 25 years.

### Main findings

**Fig. 2:**
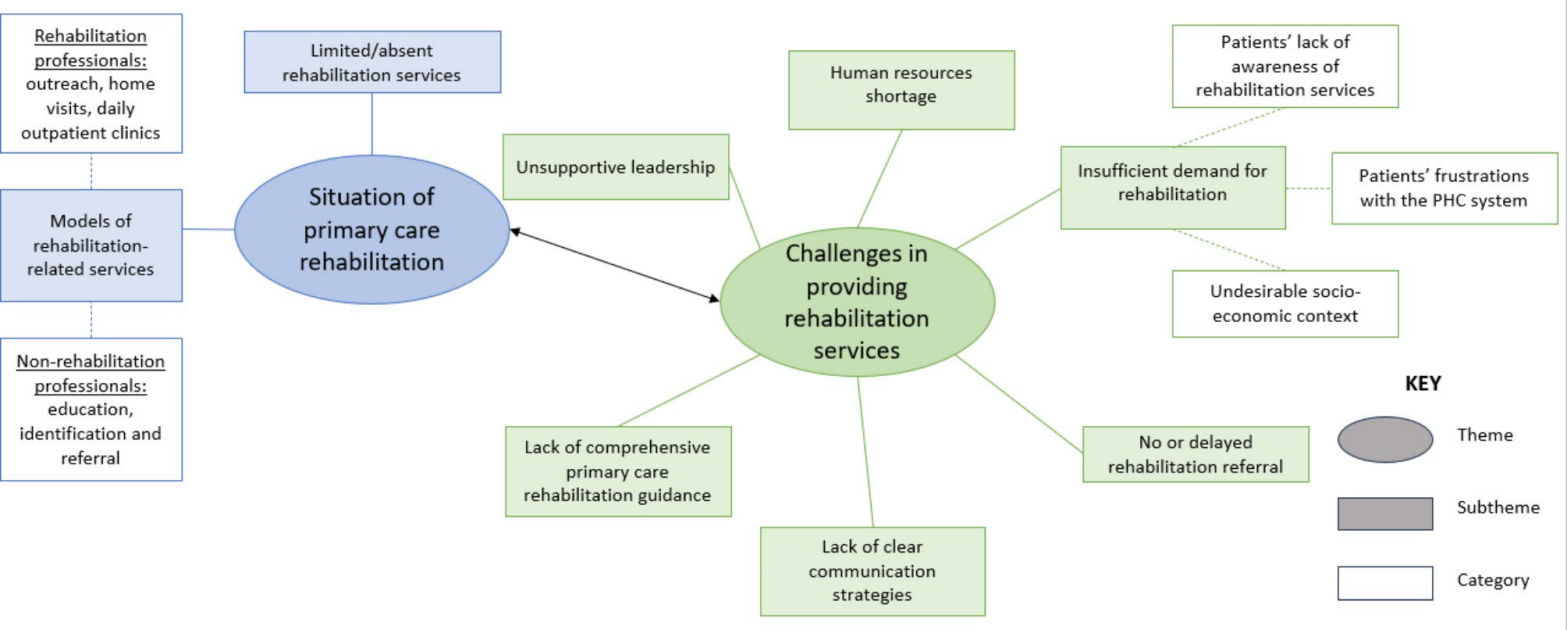
Thematic map of themes related to the study objectives and the subthemes and categories arising from participants’ responses

The two major themes were described according to the research objectives: (i) the situation of PHC rehabilitation and (ii) the challenges encountered in providing rehabilitation at PHC. The subthemes and categories were derived from an analysis of the participants’ responses. A thematic map of the themes, subthemes and categories is shown in Fig. 2.

### Situation of PHC rehabilitation

#### Inadequate/absent rehabilitation services provided by rehabilitation professionals

Although both settings were marked by serious limitations in coverage and capacity, the landscape of health-based rehabilitation services differed between the two countries’ PHC systems. In Zimbabwe, there was a lack of rehabilitation professionals at PHC facilities, with most clinics being operated by nurses without formal credentials in rehabilitation. As one participant said, *‘At the moment*,* we are only two nurses and one nurse aid’* (P8; PCN; rural; ZW). The situation was the same at the clinics in SA, but some rehabilitation professionals were employed at some of the larger community health centres. One participant listed the professions employed at one facility: *‘We don’t have a psychologist*,* we don’t have an occupational therapist*,* we don’t have a social worker…we only have physio*,* the nurses and the doctors*,* dental services*,* and youth’* (P17; PCN; urban; SA).

#### Models of rehabilitation-related services provided by rehabilitation professionals

In Zimbabwe, outreach services at the PHC facilities, previously provided by RTs from district hospitals, have been discontinued for at least five years. Infrequently, RTs assisted community health nurses when conducting individual home visits in urban areas, but this seemed to be the initiative of the community health nurses, as expressed by one participant who said, ‘*the community nurses who do home visits*,* they are the ones who have to call the rehab and work together*’ (FGD 2; PCN; urban; ZW).

South Africa reported a few models of delivering rehabilitation services, but there were notable differences between urban and rural areas. One urban community health centre offered daily outpatient rehabilitation services, but not all rehabilitation disciplines were offered.

*‘…so*,* there’s only one physiotherapist within the facility and no other rehabilitation specialist*,* and in the case where something is out of my scope when needing other services*,* I would have to refer to the nearest tertiary hospital…when I’m away there are no physiotherapy services.’* (P16; PHC PT; urban; SA).

In SA’s rural remote areas, rehabilitation professionals were mostly found at district hospitals. These professionals conducted outreach visits and home visits to selected satellite clinics.

*‘We also do home visits and then we have one outreach clinic now that we visit once a month… two outreach clinics at the moment per month for our audiologist*,* she also has an outreach day*,* so like three clinics basically a month.’* (P22; district hospital OT; rural; SA).

### Models of rehabilitation-related services provided by nonrehabilitation professionals

In both countries, the nonrehabilitation PCPs provided patient counselling and education, and referred patients with rehabilitation needs to attend higher-level or private institutions if rehabilitation services were not available at their PHC facilities.

*‘We do not provide rehabilitation services*,* but we refer them to the next level because we’re not like taught on rehabilitation. We can just assist them on how they would go to the [district] hospital and who they are going to meet at the hospital; that is how we are giving help in rehabilitation’* (P7; PCN; rural; ZW).

*‘When a patient comes to see a professional nurse*,* they educate them and tell them to do some exercises at home. For example*,* we asked them to take a walk. So*,* they do give them that education.’* (FGD 1; nursing assistant; rural; SA).

*‘If the patient is not able to walk*,* for instance*,* what we do*,* we refer so that he can get a wheelchair depending on the disability. We refer because we are a PHC*,* we don’t have anything here*,* we just refer*,* we just refer (clicking)… Even if she is not talking properly*,* we just refer so that she can meet the speech therapist in [district hospital] ….’* (P12, PCN; urban; SA).

### Challenges encountered in providing rehabilitation care at PHC

The various cadres reported several challenges in providing rehabilitation services and ensuring that patients (users) accessed and received quality rehabilitation services. Six themes were identified (Table 2).

**Table 2:** Challenges experienced in providing rehabilitation by district rehabilitation professionals providing rehabilitation at PHC, PHC rehabilitation professionals and nonrehabilitation PCPs

Subtheme	Challenges experienced by district and PHC rehabilitation professionals providing rehabilitation at PHC	Challenges experienced by nonrehabilitation PCPs
Unsupportive leadership affect resource and transport allocation	Poor awareness of rehabilitation by decision-makers affects resource allocation Difficulties procuring infrastructure, equipment, assistive technologies, and consumables	Infrastructure inaccessible to persons with disability Inadequate space at PHC facilities affecting patient confidentiality and ability to report problems
	Inadequate rehabilitation professionals at district to provide services at PHC Lack of multidisciplinary team at PHC rehabilitation departments	No rehabilitation professionals to collaborate with and refer patients to at most PHC facilities
	Lack of transport to conduct outreach visits and carry necessary equipment to outreaches	No transport to take patients to where rehabilitation services are available
Human resources shortage cause work overload and shorter patient consultations	Long patient waiting times Reduced intensity and frequency of patient treatments	Inadequate nonrehabilitation PHC workforce leading to high patient loads and limited consultation times
Lack of comprehensive PHC rehabilitation guidelines	Siloed rehabilitation training thus incompetent in other rehabilitation disciplines	Available primary care guidance focused on curative management
Inefficient referral processes	Missed/delayed/inappropriate referrals of patients	Constrained by unavailability of rehabilitation services Unaware of criteria for rehabilitation referral
Lack of clear communication strategies	Poor communication with nonrehabilitation PCPs	Poor communication/collaboration with rehabilitation professionals
	Language barriers with patients	
Insufficient demand for rehabilitation	Patients’ lack of awareness of rehabilitation services Patients’ frustrations with the PHC system The poor socioeconomic context	

#### Unsupportive leadership affected resource and transport allocation

There was no clear leadership for PHC rehabilitation. This lack of leadership left therapists without the necessary assistive technology and consumables.

*‘…the people still in charge are those nurses and maybe those doctors who might not necessarily understand what rehab needs…I think from top down to the actual workers*,* there’s no input and I think everything is thought of from a perspective of people who have no experience of what is actually happening and so they either have their own perceptions or…little knowledge then we see it kind of spirals down to equipment that’s provided or when it’s not provided and the prioritization of services and finances’* (P16; PHC PT; urban; SA).

Management’s limited understanding of rehabilitation affected human resource allocation, as explained by one participant: *‘There are… healthcare professionals sitting at home waiting to get jobs… for posts where there is a demand*,* but they are not taking out the money’* (P18; medical officer; urban; SA). The lack of rehabilitation at most PHC facilities precluded many providers from experiencing the benefits of rehabilitation and contributed to their lack of understanding of rehabilitation. One district hospital nursing manager explained, *‘…we don’t think of them sometimes*,* but if they are near*,* you can see this thing needs rehabilitation’* (P4; DNO; urban; ZW). Despite the inclusion of rehabilitation budgets at the facility level, the outcomes of these requests were unknown and not followed up. The same district hospital nursing manager admitted that she was *‘not sure about their [rehabilitation budget plan] follow-ups*,* if what they are getting is enough or are they getting what they have planned but what I know is they are included in the budget’* (P4; DNO; urban; ZW).

Similarly, the lack of prioritization of rehabilitation resulted in transport difficulties for both rehabilitation professionals and patients. In most South African rural settings, rehabilitation services were delivered via outreach from higher levels of care, such as district hospitals. However, the therapists were not supported with transport for conducting regular PHC outreach activities. In some cases, outreach visits to some PHC facilities had to be discontinued due to lack of transportation.

*‘Another big obstacle and it comes back to financing*,* but it also comes back to governance and leadership*,* is that we really struggle with transport…Often*,* administration is prioritized; rehab tends to fall pretty low down on the list for transport’* (P22; district hospital OT; rural; SA).

*‘We did do outreaches about two or three years ago when I first started working and then there were just a few problems with accessing vehicles and I think it was more institutional problems…’* (P16; PHC PT; urban; SA).

In instances where therapists were able to access transport, the transport was inappropriate for the equipment and consumables needed to deliver appropriate care.

*‘There can be some challenges to carrying out services on outreach like*,* for example*,* if a patient is needing a wheelchair repair and they took it to the clinic for that day and we didn’t bring every single one of our tools to be able to repair the wheelchair*,* then there would be a limitation on that front’* (P24; district hospital SLT; rural; SA).

In Zimbabwe, patients were often unable to access rehabilitation at higher levels of care since transport was not provided.

*‘…especially if they tell you that they do not have money to go to the hospital. Long back we used to call ambulances…those are the main challenges because we will also not have the means to transport the clients to the hospital’* (P9; PCN; rural; ZW).

#### Human resource shortages cause work overload and shorter patient consultations

Rehabilitation is delivered in a person-cantered manner and involves individualized assessment and management. The participants revealed that the time available to manage the heavy patient load was insufficient to explore rehabilitation needs. Often, patients and PCPs only explored the main reason for the visit due to time limitations.

*‘… you end up not really having enough time with the patient who patient that you can even ask how is home*,* how are you are staying at home…? You know*,* you end up just targeting what the patient has presented with… but actually most of their problems may be social problems at home of which we won’t have time to explore every complaint’* (FGD1; PCN; urban; ZW).

The PCPs experienced burnout due to staff shortages. Providers admitted to prioritizing survival over quality of life (which is the crux of rehabilitation) in their management approach to cope with high demands of care.

*‘When we are short staffed and there are competing programs*,* there is a tendency to weigh…which one is more important than the other*,* looking at which one has the capacity to end someone’s life… looking at survival’* (P8; PCN; rural; ZW).

The effects of staff shortages were also evident in the PHC rehabilitation departments in SA. Patients were often placed on a waiting list and did not receive the prescribed frequency and intensity, affecting rehabilitation quality and outcomes.

*‘One of the things that my people have been complaining about…say the dates are very far. You find the person is sick now [June]; he will get a date for next year. So*,* it is like an insult to them…you will find that they don’t even go when it comes because they have been waiting for so long*,* so they miss the date’* (P14; PCN; urban; SA).

#### Lack of clear communication strategies

In most cases, rehabilitation was delivered at higher levels of care (at district hospitals) or – in a few instances – at some larger PHC facilities (community health centres). The PCNs perceived a disconnect between the available rehabilitation professionals and themselves. One participant described how *‘rehabilitation is not something that is very popular like other departments [that] work hand in hand close*,* close*,* close… the rehabilitation seems far away’* (P4; DNO; urban; ZW). The poor collaboration resulted in a lack of familiarity and understanding about the roles and contributions of the different rehabilitation disciplines and may have resulted in decreased referral of patients for appropriate care.

In SA’s urban and rural contexts, poor communication also existed between the PCPs and the patients because of varied foreign languages, local languages, and dialects.

*‘[T]he language barrier is one of the things that makes difficult to even take history from the client…you have got people from other countries…others are…from the upper parts of South Africa’* (P14; PCN; urban; SA).

*‘[T]he majority of our clinical team and the majority of our rehab team are English speakers*,* and I think 98% of our population in the sub-district are isiXhosa speaking and so that poses a significant barrier to providing quality rehab.’* (P22; OT district hospital OT; rural; SA).

#### Lack of comprehensive PHC rehabilitation guidelines

The lack of evidence-based guidance compounded by the prevailing siloed university rehabilitation training resulted in fragmented rehabilitation service provision.

*‘…the exposure at the varsity [university] level to other rehab professions is quite limited’*, making it difficult *‘to understand some of the speech terms or some of [the] management options’* (P22; district hospital OT; rural; SA).

In both countries, the available primary care guidance focused on curative management. Many nonrehabilitation PCPs were familiar with some PHC guidelines, such as the Essential Drug List (EDL), Adult Primary Care (APC) or condition-specific guidelines. However, similar practical rehabilitation guidance was not available; consequently, rehabilitation was often not considered.

*‘The [guidelines] are very limited. We have guidelines on the management of hypertensive clients [and] the management of our diabetic clients. But what is lacking is training’* (P8; PCN; rural; ZW).

*‘At times you will find that there was no need for medication or a pill but just to tell that person what to do at home… because when you say you’ve got a pain here*,* what I think is Panado*,* is Brufen to give you because it’s something that will relieve the pain*,* there is nothing I would do*,* say you can exercise and do this*,* there is no such…’* (P14; PCN; urban; SA).

The PCPs highlighted their adaptability and commitment to lifelong learning if guidelines and training were made available.

*‘As nurses*,* we are very flexible. Like at the clinic*,* we do most of the work*,* so if we can do maybe a short course*,* it would be good for us to help the minor cases*,* then we refer severe cases’* (FGD1; PCN; urban; ZW).

#### No or delayed rehabilitation referral

Inadequate referral to rehabilitation was evident in both countries. The absence of rehabilitation services or specific rehabilitation disciplines at PHC constrained PCPs’ capacity to refer rather than indicating a deliberate decision to withhold referrals. PCPs commonly referred patients requiring rehabilitation to doctors at higher levels of care first, potentially lengthening the care pathway for these patients.

*‘Since there are not really places where you can refer the clients for speech therapy and all*,* we don’t usually refer them. We just assist the client in whatever they want*,* and then they come for their next appointment’* (P13; PCN; urban; SA).

*‘We don’t directly refer to them to rehabilitation or even if you see that this condition need rehabilitation. We first refer to a doctor. So*,* it’s referral to a higher level. Then the higher level will decide if the patient needs rehabilitation.’* (P2; PCN; urban; ZW).

The curative approach to management may explain the delayed referrals as suggested by one participant.

*‘[laughter]. For us*,* pain management is a painkiller. Ya*,* I’m being honest with you…Then*,* if that person comes back frequently that is when we would say*,* this backache I think needs further management’* (P9; PCN; rural; ZW).

Furthermore, some PCPs had insufficient knowledge and awareness of the scope of rehabilitation to adequately identify and refer patients with rehabilitation needs as realized by one participant in SA: ‘*I need to look into people who would need it [rehabilitation] even if I don’t think so…and then find out how do we refer and how do we get these services to them’* (P19; PCN; urban; SA). Similarly, the DNO in Zimbabwe observed: *‘There are some gaps because the rehabilitation personnel are the ones who are experts in this area but it’s the nurses who identify and then refer them…They [nurses] don’t have knowledge that would help them to identify the patients and also to help them if they identify a problem who they need to consult’* (P4; DNO, urban, ZW).

The rehabilitation PCPs confirmed the inappropriate rehabilitation referrals, *‘…very often they don’t know what they’re referring for and you will often get someone referring a patient who had a stroke ten years ago… [need to] inform about referral patterns and the referral pathways’* (P16; PHC PT; urban; SA).

#### Insufficient demand for PHC rehabilitation

The time, resources, and effort spent on conducting outreach visits to the clinics in SA were wasted when few patients attended the scheduled clinics. This resulted in some outreach services being discontinued, as the district rehabilitation professionals decided that they were *‘not really utilizing [their] time very well in going to outreach’* (P24; district hospital SLT; rural; SA).

The participants reported on three factors that hindered patients from seeking rehabilitation in the various contexts:

(i) Patients’ lack of awareness of rehabilitation services

Patients’ lack of awareness about rehabilitation resulted from poor health literacy, cultural beliefs and interrelated myths and misconceptions. Examples of such beliefs were given:

*‘If someone has a stroke*,* they… [think] that the person is bewitched*,* that is why they are no longer talking or they are no longer walking’* (P7; PCN; rural; ZW).

*‘[T]here are a lot of mental healthcare users that don’t present to the hospital or clinic because there’s some entrenched ancestral*,* witchcraft*,* traditional ideas about what mental health or mental illness is and what it means and how it’s to be treated…doesn’t quite fit with being treated with western medicine and so the route that patients often take then is in traditional help through a sangoma or traditional medicine’* (P22; district hospital OT; rural; SA).

Thus, in both settings, it was common for patients to seek alternative healthcare from traditional healers or faith healers.

(ii) Patients’ frustrations with the PHC system

Prior negative experiences when attending PHC deterred some patients from seeking further healthcare. One CHW in SA highlighted some healthcare system challenges, including overcrowded clinics and inefficient appointment systems:

*‘Sometimes clients block me not to go to their houses. Some are not welcoming at all. Some would tell me straight in the eyes that they are tired of me coming to their houses*,* telling them to go to the clinic where people must sit the whole day before they can be attended to’* (P26; CHW; rural; SA).

(iii) Undesirable socioeconomic context.

The undesirable socioeconomic context caused patients to prioritize their basic survival over obtaining healthcare and this challenge seemed to be more acute in rural settings. Most individuals in SA’s rural communities depended on social assistance from the government and NGOs for their subsistence, which was insufficient to cover the additional healthcare costs of accessing rehabilitation. To describe the situation, one participant said,

*‘Some of them mention that they… do not have anything to eat… Some are not working because they do not even have an ID*,* but they are in their 30s and 40s… and have no parents… you see the situation is dire’* (P26; CHW; rural; SA).

Poverty in Zimbabwe’s rural communities similarly hindered patients’ access to rehabilitation when referred to higher levels of care.

*‘Most of the community*,* they are peasant farmers and all*,* so…if you refer them*,* they talk about the challenge of money’* (P6; PCN; rural; ZW).

Patients may have valued rehabilitation, but financial concerns contributed to patients’ hesitation or inability to seek these services.

## Discussion

Based on the articulated perceptions of PCPs and managers in SA and Zimbabwe, this qualitative study revealed the current situation of PHC rehabilitation, and the challenges experienced by PCPs in the provision of PHC rehabilitation services. The current PHC rehabilitation services in the two low-resource contexts were perceived as severely lacking, and several challenges related to the prevailing dire situation were raised.

The description of current rehabilitation services at PHC indicates that the models of service delivery provided by rehabilitation PCPs include out-patient clinic visits, outreach and home visits, which corroborates with the findings reported by Maseko et al. (2024) [14]. However, rehabilitation services in the two settings were mostly nonexistent at the PHC level, especially at the smaller satellite clinics. The absence of rehabilitation services at most PHC facilities was attributed to unsupportive leadership, which consequently resulted in shortages of the rehabilitation workforce and insufficient financial and resource allocation for PHC rehabilitation. Previous research has shown that decision makers’ lack of understanding about rehabilitation may result in not adjusting resource provision to accommodate changing methods of delivering rehabilitation services or increasing population needs [40]. Such adjustments may include providing modern equipment, continuous professional development, or expanding workforce capacity. The literature points to existing weaknesses in LMICs’ health systems that affect continuity of care [41] and negatively impact the prioritization of rehabilitation [40]. These countries experience difficulties in retaining skilled rehabilitation professionals due to economic instability, lack of incentives, and work overload, which can cause rehabilitation health professionals to migrate to the private sector or other countries [42]. Additionally, other studies have shown that the shortage of rehabilitation staff may be due to inadequate numbers of trained professionals or a lack of financing to hire rehabilitation professionals to fill vacant posts [43]. Thus, in the current absence of rehabilitation professionals, there may be a need for available nonrehabilitation PCPs to proactively provide basic rehabilitation services to underserved populations.

The nonrehabilitation PCPs focus on two aspects: patient education and the identification and referral of patients to available rehabilitation services. The significance of these activities is confirmed by a systematic review that highlighted the importance of patient education at PHC in empowering patients through self-management of functioning problems, prevention of complications and positive lifestyle changes [44]. Early identification and referral enable patients to promptly initiate rehabilitation interventions and address potential health issues before they worsen [44]. Although the currently available rehabilitation services are suboptimal, the efforts of PCPs provide a good foundation for ensuring that PCPs provide effective patient education and refer patients appropriately.

One significant challenge experienced by nonrehabilitation PCPs in providing effective patient education, adequate rehabilitation referral, and basic rehabilitation was the lack of clear rehabilitation guidance. Our findings corroborate previous evidence regarding the exclusion of rehabilitation in current South African PHC standard treatment guidelines [12]. The current study highlighted the potential for delayed or missed diagnoses of rehabilitation needs and fragmented care due to a lack of clear rehabilitation referral criteria. The lack of standardized training in rehabilitation practices led to non-evidence-based practices and varying competencies among the PCPs, which resulted in inconsistent and inadequate care. Our study showed the potential of PCPs who were willing to undertake short courses, workshops, or post-basic specialization training for basic rehabilitation provision. This aligns with the PHC-specific training needs expressed by nurses in a recent South African study by Kordom et al. (2023), which predominantly encompassed skills and management for various health conditions [45]. Thus, PCPs may be upskilled to provide more reliable and appropriate patient education related to rehabilitation as well as basic rehabilitation interventions [45]. Although the quality of rehabilitation provided may still be subpar due to a lack of financial and physical resources, the quality may be measured by the ability to meet the population’s rehabilitation needs. Thus, it is important for rehabilitation guidelines to be contextually relevant [46].

The already overburdened district hospital-based rehabilitation professionals in SA made efforts to fill the gap in the provision of PHC rehabilitation by means of outreach and home visits. Irregular outreaches were once again related to an inadequate rehabilitation workforce, and it was even more challenging to provide outreach services as a multidisciplinary team. Maseko et al. (2024) reported a decrease in outreach frequency due to costs and transport unavailability, despite efforts to schedule and peer audit these activities [17]. To offset the burden on district-level rehabilitation professionals to provide outreach PHC rehabilitation, rehabilitation may be included in PHC ward-based outreach teams that are quite active in both countries [47]. The few available district rehabilitation professionals may take on mentoring or supervisory roles with PCPs to ensure that quality rehabilitation services are provided. To further reduce the need for outreach, the training of community rehabilitation workers (CRWs) to screen, refer and provide basic PHC rehabilitation has been successfully initiated in both SA and Zimbabwe [48] but needs to be sustained. The current study’s authors suggest that another possibility exists for currently qualified rehabilitation professionals undergoing specialized training to become PHC rehabilitation specialists who have basic competency across all rehabilitation disciplines. Exposure to training within rural and underserved settings enhances cadres’ preparedness to work in these settings; thus, even community service therapists would be proficient in PHC approaches and a broader range of rehabilitation skills [49]. However, the current study revealed that even these outreach efforts were futile when patients did not demand rehabilitation services.

The current study further emphasizes the potential role that patients can play in enhancing the utilization of rehabilitation services. The re-engineering of the PHC system might not yield the desired results if the services remain underutilized. Patients need to improve their health literacy (including rehabilitation needs). This involves knowing what rehabilitation care to seek and the quality of rehabilitation services to demand. A previous study highlighted how patients must consult credible sources of information and communicate their functioning problems to receive appropriate care from PCPs [50]. Enhancing health literacy at the community level may be particularly beneficial for individuals who are not able to enter the PHC system. This can be achieved through health education or awareness campaigns [50], utilizing local media and community gatherings to promote awareness of the benefits and availability of rehabilitation services. The inclusion of community leaders has been shown to be effective in driving the implementation of new initiatives, influencing health-related behavioural change and debunking cultural stigmas and misconceptions, which negatively impact the acceptance and utilization of health services [51]. These low-cost measures will greatly improve rehabilitation in these settings. However, while our study highlighted patients’ lack of health literacy as a factor in the underutilisation of rehabilitation services, it is not the sole cause of limited demand for rehabilitation. Our previous work which engaged the PHC patients in these settings identified additional barriers including geographic accessibility, financial barriers, cultural beliefs and unclear referral pathways [52, 53]. Future studies can map patient itineraries through the healthcare system, particularly for those requiring rehabilitation, to identify bottlenecks and barriers to access at various points in the care continuum.

The grim reality is that in the current low-resource context, rehabilitation services are largely nonexistent at PHC. Consequently, before entering the discourse about strengthening rehabilitation, there is a need to *establish* basic rehabilitation services. More importantly, all PHC systems must be fortified to have the capacity to deliver effective rehabilitation services; rehabilitation cannot be scaled up in isolation [54]. Planning for rehabilitation services in LMICs cannot follow a one-size-fits-all approach, as there were notable differences between these settings. The current functionality of PHC systems varies significantly, largely due to the countries’ different social, economic, and political climates. SA seems to be a few steps ahead in the provision of rehabilitation at PHC (such as active outreach programs, better infrastructure, and more PCPs) but not without its challenges. Most challenges hinge upon inadequate financial resources for rehabilitation, and thus, potential strategies will only be possible with political will and financial investment. This will require advocacy so that decision-makers acknowledge the value of rehabilitation. Both settings apply a similar approach to strengthening PHC by providing an essential package of healthcare, of which rehabilitation needs to be part [55]. Due to resource constraints, the initial focus of integrating rehabilitation at PHC may be on providing basic rehabilitation services rather than comprehensive care that involves the full range of rehabilitation services.

The generalizability of the findings to other contexts may be limited, as the status of rehabilitation service delivery and available resources for enhancing rehabilitation may be different in other districts or provinces. However, the comprehensive description of the current study contexts allows readers to assess its applicability to other settings. Moreover, our study had limited representation from the managers in the selected settings. Quantitative assessments of rehabilitation provision and comprehensive analyses of health policies at PHC may be conducted, particularly in Zimbabwe where such studies have not been identified, to confirm or build on the findings of this study. Further work should consider the development of feasible strategies that address the existing gaps and challenges in the provision of PHC rehabilitation cited in this study.

## Conclusion

Significant gaps and challenges exist in rehabilitation service provision at PHC in SA and Zimbabwe. Unsupportive leadership, inadequate financial and human resources, and limited understanding of rehabilitation were the main challenges and will continue to pose a risk to the successful integration of rehabilitation at PHC in low-resource settings. It may be possible for both settings to maximize the currently available human and physical resources to provide basic rehabilitation services that meet the population needs. The current role of nonrehabilitation PCPs in patient education, identification, and referral may be strengthened through improved collaboration with available rehabilitation professionals, the development and implementation of rehabilitation-specific PHC guidelines and ongoing training opportunities. However, contextualized rehabilitation guidelines are urgently needed to make such improvements possible. Meanwhile, advocacy is required for financial investment in establishing basic PHC rehabilitation services.

## Supplementary Information

Supplementary Material 1.

## Data Availability

The datasets generated during and/or analysed during the current study are available in the SUNScholarData repository at https://doi.org/10.25413/sun.23545428.
