# Supplementary material for: A qualitative study on rehabilitation services at primary health care: insights from primary health care stakeholders in low-resource contexts
Source: BMC Health Serv Res. 2024 Oct 23;24:1272. doi: 10.1186/s12913-024-11748-9 (PMC11515710; doi:10.1186/s12913-024-11748-9)
Supplement: Supplementary file 1 — Supplementary Material 1. [file 12913_2024_11748_MOESM1_ESM.pdf]

Interview Guide – PHCP

(Focus of interview: To explore PHCPs' perceptions of current rehabilitation service delivery (e.g., mode and shortcomings) based on the rehabilitation service needs of adults presenting at primary care level and explore how suggested innovations to improve rehabilitation at primary care may be integrated into the care pathways)

Please help me better understand your perspectives regarding current rehabilitation service delivery and provide suggestions on ow we can strengthen rehabilitation at primary care.

How often do you provide a rehabilitation service at this clinic/ CHC?

What care do you provide to patients with functioning problems during consultations? What rehabilitation services do you provide (at PHC)? What modes?

Why do you think rehabilitation is important for patients in the community?

What do you think patients are concerned about when they report functioning problems to you? What is important to them?

What challenges are you currently facing in delivering rehabilitation services to patients at primary care?

In your opinion, what needs to be done by primary health care workers to improve rehabilitation of patients with functioning problems and underlying impairments?

In your opinion, what needs to be done by patients to improve rehabilitation of their functioning problems and underlying impairments?

What new methods of delivering rehabilitation would work in your setting?

Ending question: Is there anything else you which I haven't asked you which you would like to share with me?

Closure of Interview

Thank you for your time and for sharing your experiences with us.

I will stop the recording now.

My reflections on the interview process:
